# Supplementary material for: A Transparent Hydrogel-Ionic Conductor with High Water Retention and Self-Healing Ability
Source: Materials (Basel). 2024 Jan 6;17(2):288. doi: 10.3390/ma17020288 (PMC10817594; doi:10.3390/ma17020288)
Supplement: Supplementary file 1 [file materials-17-00288-s001.zip › materials-2774469-supplementary.pdf]

Supplementary Information

A Transparent Hydrogel-Ionic Conductor with High Water Retention and Self-Healing Ability

Yangwoo Lee [1], Ju-Hee So [2,*] and Hyung-Jun Koo [1,*]

[1] Department of Chemical & Biomolecular Engineering, Seoul National University of Science & Technology, 232 Gongneung-ro, Nowon-gu, Seoul 01811, Republic of Korea; diddnqkqh@gmail.com
[2] Material & Component Convergence R&D Department, Korea Institute of Industrial Technology, Ansan 15588, Republic of Korea
*Correspondence: jso@kitech.re.kr (J.-H.S.); hjkoo@seoultech.ac.kr (H.-J.K.)

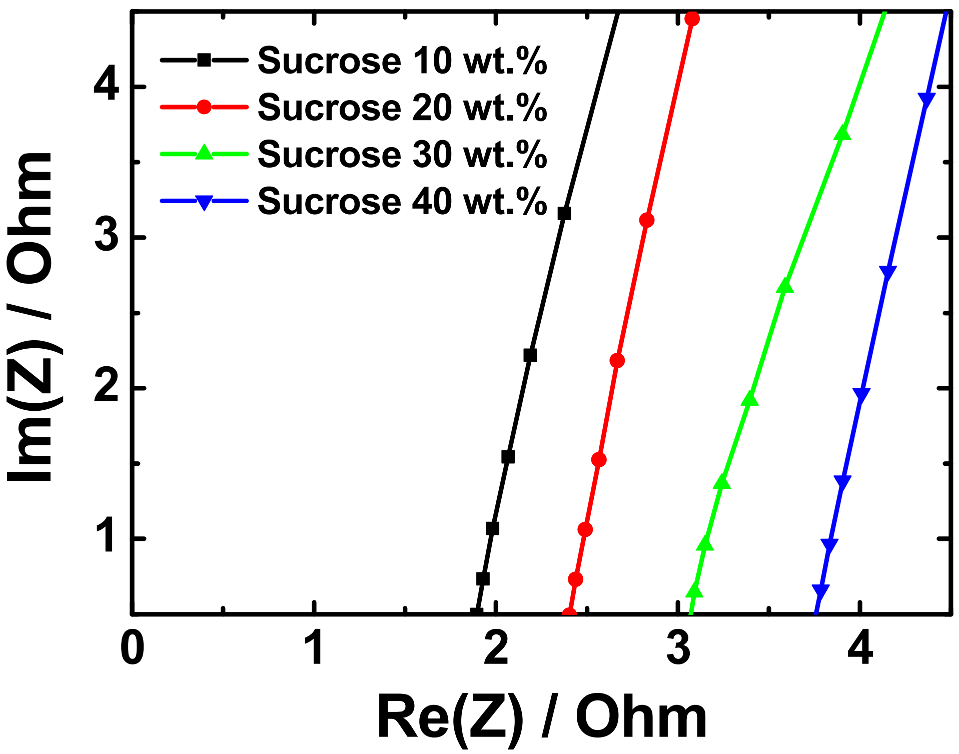

Supplementary Figure S1. Nyquist plots of NaCl–Suc/A hydrogels with different sucrose concentrations. The concentration of NaCl is 2M. AC frequency range is 0.1 Hz–1000 kHz.

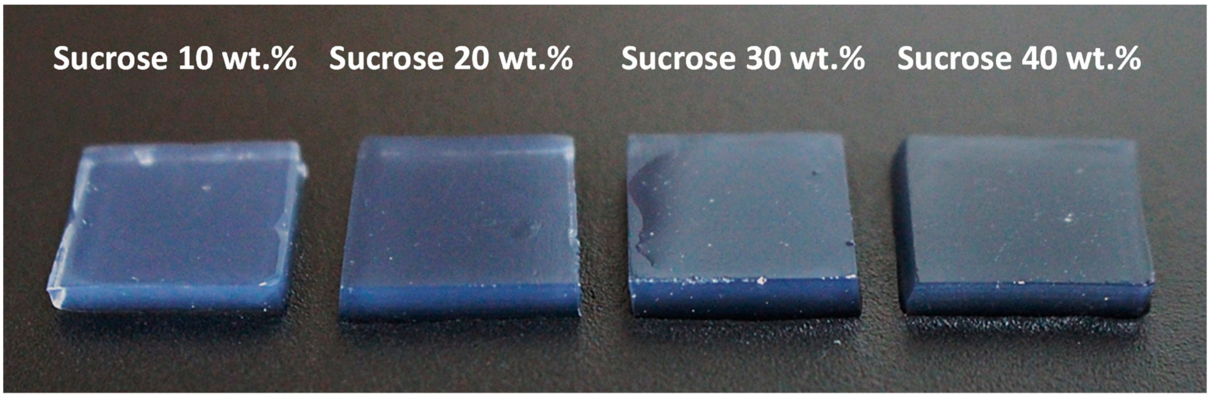

Supplementary Figure S2. Photograph of as-prepared NaCl–Suc/A hydrogels with different sucrose concentrations.
